# Expect the Unexpected: Frequency, Predictors, and Survival Impact of Pathological Upstaging from Non-Muscle-Invasive to Muscle-Invasive Bladder Cancer Following Radical Cystectomy

**DOI:** 10.3390/cancers18111733

**Published:** 2026-05-26

**Authors:** Federico Ceria, Gad Muhammad, Francesco Del Giudice, Youssef Ibrahim, John O’Kelly, Ramesh Thurairaja, Rajesh Nair, Elsie Mensah, Muhammad Shamim Khan, Yasmin Abu Ghanem

**Affiliations:** 1 Guy’s and St. Thomas’ NHS Foundation Trust, Guy’s Hospital, London SE1 7EH, UK; f.ceria@studenti.unisr.it (F.C.); m.gad@nhs.net (G.M.);; 2 Department of Urology, University Vita Salute, San Raffaele Hospital, 20132 Milan, Italy; 3 Department of Maternal-Infant and Urological Sciences, “Sapienza” University of Rome, Umberto I Hospital, 00185 Rome, Italy; francesco.delgiudice@uniroma1.it; 4 Department of Urology, Stanford University School of Medicine, Stanford, CA 94304, USA

**Keywords:** bladder cancer, non-muscle-invasive, radical cystectomy, pathological upstaging, carcinoma in situ, variant histology, urethral involvement, survival outcomes, neoadjuvant chemotherapy

## Abstract

**Background and Objectives:** Pathological upstaging from non-muscle-invasive bladder cancer (NMIBC) to muscle-invasive disease (MIBC) at radical cystectomy (RC) compromises preoperative risk stratification and may deprive patients of the survival benefit conferred by neoadjuvant chemotherapy. This study aimed to define the frequency and independent predictors of pathological upstaging in a contemporary single-institution cohort, and to characterise its impact on recurrence-free, disease-specific, and overall survival. **Materials and Methods:** We conducted a retrospective review of a prospectively maintained database of all patients who underwent RC and pelvic lymphadenectomy for urothelial bladder cancer at our institution between January 2009 and December 2023. Upstaging was defined as the final pathological stage ≥ pT2 or pN+ from a clinical stage of <T2N0M0. Clinicopathological factors were evaluated for their association with upstaging using chi-squared, Fisher’s exact, and logistic regression analyses. Survival outcomes—recurrence-free survival (RFS), disease-specific survival (DSS), and overall survival (OS)—were estimated by the Kaplan–Meier method and compared using the log-rank test. Multivariable Cox proportional hazards regression identified independent prognostic factors. **Results:** Of 1002 patients who underwent RC during the study period, complete clinicopathological data were available for 826. Primary clinical stage at presentation was MIBC (≥T2) in 448 (54.2%) and NMIBC (<T2) in 378 (45.8%). Among NMIBC patients, 102 (27.0%) were upstaged to MIBC at final pathology. Multivariable logistic regression identified concomitant carcinoma in situ (CIS) (*p* = 0.042), variant histology—predominantly squamous differentiation (*p* = 0.003)—and urethral involvement (*p* = 0.003) as independent predictors of upstaging. Upstaged patients had significantly worse 5-year RFS (*p* < 0.001), DSS (*p* = 0.01), and OS (*p* < 0.001) compared with patients who remained NMIBC. No statistically significant difference in survival was observed between patients upstaged at RC and those presenting with primary MIBC. **Conclusions:** Pathological upstaging occurs in more than a quarter of patients undergoing RC for NMIBC and confers a survival penalty equivalent to that of primary MIBC. Concomitant CIS, variant histology, and urethral involvement identify those at highest risk. These patients warrant aggressive preoperative counselling, expedited surgical planning, and consideration of perioperative systemic therapy.

## Simple Summary

Accurate staging before radical cystectomy has direct implications for treatment decisions and patient outcomes. We reviewed 826 patients with bladder cancer who underwent radical cystectomy at our institution between 2009 and 2023, asking how frequently patients staged as non-muscle-invasive bladder cancer (NMIBC) were found to harbour muscle-invasive disease (MIBC) at final pathology, and which tumour and patient characteristics drive this risk. Approximately one in four such patients (27%) was upstaged to MIBC, and upstaged patients suffered substantially worse oncological outcomes than those who remained NMIBC. Concomitant carcinoma in situ (CIS), variant histological subtypes—in particular squamous differentiation—and urethral involvement were the strongest independent predictors of upstaging. The survival profile of patients upstaged at cystectomy closely resembled that of patients who presented with primary MIBC, suggesting that understaging carries the same oncological penalty as overt muscle-invasive disease. These findings support thorough preoperative risk stratification and earlier surgical intervention in high-risk NMIBC.

## 1. Introduction

Clinical staging of bladder cancer is wrong often enough to matter. Concordance rates between clinical and final pathological stage range from as low as 20% to approximately 80%, with most discordances involving under-staging [1,2]. The consequences are most acute when muscle invasion goes undetected: a patient managed as T1 high-grade may harbour pT2 or even pT3 disease, with profound implications for the timing of surgery and the administration of neoadjuvant cisplatin-based chemotherapy (NAC), which confers a survival benefit in cT2–4a disease [3,4].

Bladder cancer accounts for approximately 573,000 new cases and 213,000 deaths annually worldwide [5]. In the United Kingdom, it is the fourth most common cancer in men and carries a disproportionate economic burden relative to its incidence, largely owing to the intensive surveillance protocols required for non-muscle-invasive disease [6,7]. Approximately 70–75% of new diagnoses present as non-muscle-invasive bladder cancer (NMIBC), encompassing disease confined to the urothelium (Ta, carcinoma in situ [CIS]) or the lamina propria (T1); the remaining 25–30% are muscle-invasive at first presentation [8].

The management of NMIBC hinges on transurethral resection of the bladder tumour (TURBT), supplemented in high-risk disease by intravesical Bacillus Calmette–Guérin (BCG) immunotherapy [9,10]. Radical cystectomy (RC) is reserved for patients with muscle-invasive disease or for those with high-risk or very-high-risk NMIBC in whom conservative measures have failed—including BCG-unresponsive disease, persistent high-grade T1 tumours, and multifocal disease refractory to intravesical therapy [11]. Both the European Association of Urology (EAU) 2025 NMIBC guidelines and the American Urological Association (AUA)/Society of Urologic Oncology (SUO) 2024 amendment concur on the indications for early cystectomy, and specifically acknowledge variant histology and urethral involvement as features that should prompt consideration of immediate RC [12,13].

Pathological upstaging—defined as the finding of ≥pT2 or pN+ disease at RC in a patient clinically staged as NMIBC—has been described in 20–50% of patients in historical series [14,15]. The wide variation reflects heterogeneity in patient populations, institutional TURBT quality, re-resection practices, and the threshold for proceeding to RC. Several clinical and pathological factors have been implicated in upstaging risk, including concomitant CIS, T1 substage, variant histological subtypes, lymphovascular invasion (LVI), and hydronephrosis [16,17,18]. Data from the International Robotic Cystectomy Consortium (IRCC) confirmed that upstaged patients experienced substantially worse recurrence-free, disease-specific, and overall survival compared with those who remained NMIBC [18].

Most published series are limited by relatively small sample sizes, heterogeneous surgical approaches, or a failure to report survival outcomes stratified by initial clinical stage. The biological drivers of upstaging—particularly the role of specific variant histologies and urethral involvement—remain incompletely characterised in contemporary robotic cystectomy cohorts. Emerging data also suggest that molecular subtyping may further refine risk stratification: non-luminal tumours, identified using genomic subtyping classifiers, demonstrate significantly higher rates of upstaging than luminal counterparts, even after adjustment for clinical variables [19].

The present study sought to address several of these gaps by analysing a large, contemporary single-institution RC database spanning 14 years. Our objectives were threefold: to determine the frequency of pathological upstaging in the context of modern robotic-assisted RC; to identify independent clinicopathological predictors of upstaging using multivariable modelling; and to compare survival outcomes across three clinically meaningful groups—patients with primary MIBC, patients upstaged from NMIBC, and patients who remained NMIBC at final pathology.

## 2. Materials and Methods

### 2.1. Study Design and Patient Selection

We conducted a retrospective analysis of a prospectively maintained institutional RC database. All patients who underwent RC and pelvic lymphadenectomy for urothelial carcinoma of the bladder at our centre between January 2009 and December 2023 were eligible for inclusion. Patients were excluded if they had a non-urothelial primary histology, underwent salvage RC following prior definitive radiotherapy, or had incomplete pathological staging data. In total, 176 patients were excluded (115 for non-urothelial histology or salvage indication; 61 for incomplete data), leaving 826 patients available for analysis.

Upstaging was defined as a final pathological stage of ≥pT2 +/− pN+ in a patient whose pre-cystectomy clinical stage was <T2N0M0 (i.e., cTa, cT1, or cTis, all with N0M0). Lymph node positivity (pN+) alone, in the absence of ≥pT2 disease, was not used as a standalone criterion for upstaging in the primary analysis. Patients were subsequently divided into three analytical cohorts: (1) primary MIBC—those with clinical ≥ cT2 disease at presentation; (2) upstaged NMIBC—those with clinical <T2 disease who were found to have ≥pT2 or pN+ at final pathology; and (3) non-upstaged NMIBC—those with clinical <T2 disease who remained pathologically non-muscle-invasive. As this study was retrospective and involved no intervention or direct contact with participants, ethical approval was not required.

### 2.2. Clinicopathological and Operative Variables

Patient-level data recorded included age at diagnosis and at cystectomy, sex, ethnicity, body mass index (BMI), and American Society of Anaesthesiologists (ASA) physical status score. Tumour-related variables included clinical stage and grade before RC (based on TURBT pathology and cross-sectional imaging), the presence of concomitant CIS, variant histological subtypes (squamous, sarcomatoid, micropapillary, glandular, small cell/neuroendocrine, plasmacytoid), lymphovascular invasion (LVI), urethral involvement, and ureteric invasion. Operative data included surgical approach (open, robotic-assisted [RARC], or laparoscopic), extent of lymphadenectomy, and type of urinary diversion. Pathological stage was assigned according to the 2017 TNM classification system (8th edition).

### 2.3. Preoperative Assessment and Surgical Technique

All patients underwent TURBT at our institution or at referring units, in the latter case, with mandatory review of pathological slides or repeat TURBT at our centre. Bimanual examination under anaesthesia was performed in all cases. Cross-sectional staging with contrast-enhanced CT of the chest, abdomen, and pelvis was obtained routinely; MRI was used selectively, principally for surgical planning in patients with MIBC or where CT findings were equivocal. All specimens were reviewed by dedicated genitourinary pathologists. Re-TURBT was performed at the discretion of the treating surgeon, particularly for cases with T1 high-grade disease, absent detrusor muscle in the initial specimen, or suspected incomplete resection.

RC was performed by total bladder excision with distal ureterectomy; in males, the prostate and seminal vesicles were removed unless nerve- or prostate-sparing techniques were employed; in females, anterior exenteration including hysterectomy, bilateral salpingo-oophorectomy, and anterior vaginal wall excision was standard unless organ preservation was deemed oncologically and technically appropriate. Pelvic lymphadenectomy included at minimum the obturator, external iliac, and internal iliac nodal packets; extended dissection to the common iliac vessels and presacral region was performed at the surgeon’s discretion.

### 2.4. Postoperative Follow-Up

Postoperative surveillance was conducted according to EAU guidelines: cystoscopy and upper tract imaging every three months for the first year, every six months until year five, and annually thereafter. Follow-up was defined from the date of RC to the date of last clinical contact, death, or disease recurrence, whichever occurred first.

### 2.5. Statistical Analysis

Categorical variables were compared using the chi-squared test or Fisher’s exact test as appropriate. Continuous variables were summarised as median with interquartile range (IQR) and compared using the Mann–Whitney U test. Logistic regression was used to identify factors independently associated with pathological upstaging; variables with *p* < 0.10 on univariable analysis were entered into the multivariable model. Kaplan–Meier survival curves were constructed for RFS, DSS, and OS, and group comparisons were made using the log-rank test. Multivariable Cox proportional hazards models were built to identify independent predictors of survival endpoints; results are reported as hazard ratios (HRs) with 95% confidence intervals (CIs). Temporal trends in upstaging rates were assessed using the Cochran–Armitage trend test. All tests were two-sided, with statistical significance set at α = 0.05. Analyses were performed using SAS version 9.4 (SAS Institute, Cary, NC, USA), R version 4.3.1, and IBM SPSS Statistics version 27.0.

## 3. Results

### 3.1. Cohort Characteristics

Of the 826 patients included, the median age at cystectomy was 70.2 years (IQR 62.9–75.8), and 598 (72.5%) were male. The majority self-identified as White/Caucasian (72.8%), with smaller proportions identifying as Mixed (6.3%), Black (4.2%), and Asian (2.2%). The median BMI was 27.1 (IQR 23.9–30.5) and the median follow-up was 24 months (IQR 12–48). Most patients had an ASA score of 1–2 (70.3%). The predominant surgical approach was RARC (70.0%), followed by open RC (approximately 30%), with two patients undergoing laparoscopic cystectomy. The majority of patients received an ileal conduit diversion (approximately 90%).

Primary clinical stage at presentation was MIBC (≥cT2) in 448 patients (54.2%) and NMIBC (<cT2) in 378 (45.8%). Among the latter, 102 (27.0%) were upstaged to MIBC at final pathology. Final pathological stage distribution across the full cohort is detailed in Table 1.

Pathological stage distributions after RC, calculated among the 782 patients with complete T-stage data, were as follows: pT0/pTx 15.2%, pTis 11.9%, pTa 8.1%, pT1 12.8%, pT2 16.4%, pT3 28.4%, and pT4 7.3%. Concomitant CIS was detected in 286 patients (34.6%). Among the 698 patients with recorded histological subtype data, 590 (84.5%) had pure urothelial carcinoma and 108 (15.5%) demonstrated variant histology. The frequency of variants was: squamous differentiation (N = 49, 7.0%), sarcomatoid (N = 14, 2.0%), micropapillary (N = 13, 1.9%), glandular (N = 13, 1.9%), small cell/neuroendocrine (N = 10, 1.4%), plasmacytoid (N = 5, 0.7%), and adenocarcinoma (N = 4, 0.6%). Positive surgical margins and ureteric invasion were each detected in 56 patients (6.8%).

### 3.2. Comparison of NMIBC and Primary MIBC Cohorts

When comparing baseline characteristics between the NMIBC and primary MIBC groups (Table 2), both cohorts were broadly similar with respect to age, sex, ethnicity, BMI, ASA score, surgical approach, and referral pattern. Preoperative MRI was performed significantly more often in MIBC patients, reflecting its role in surgical planning rather than staging (42.7% vs. 25.2%, *p* = 0.002). Concomitant CIS was substantially more prevalent in the NMIBC group (74.1% vs. 46.9%, *p* < 0.01), while variant histology was more frequent among those presenting with primary MIBC (20.8% vs. 9.0%, *p* < 0.01). These differences likely reflect the distinct biological trajectories of these two disease states.

### 3.3. Predictors of Upstaging: Upstaged vs. Non-Upstaged NMIBC

The upstaged and non-upstaged NMIBC groups were well matched for age, BMI, ASA score, surgical approach, and rates of prior intravesical therapy (Table 3). However, upstaged patients were significantly more likely to be female (36.3% vs. 25.0%, *p* = 0.02) and to identify as Black (9.2% vs. 4.7%, *p* < 0.05). The clinical significance of these demographic associations is discussed further below.

On pathological review, upstaged patients were substantially more likely to harbour variant histology (20.6% vs. 3.3%, *p* < 0.01) and urethral involvement (20.0% vs. 3.3%, *p* < 0.01). Additionally, cT1 disease was more prevalent in the upstaged group compared with cTa disease (69.6% vs. 55.1%, *p* = 0.013), consistent with the known understaging risk inherent to lamina propria-invasive tumours. The rate of concomitant CIS was similar between upstaged and non-upstaged NMIBC groups on univariate analysis (65.6% vs. 76.0%, *p* = 0.84), indicating that CIS prevalence alone did not distinguish those who were upstaged. Rates of prior intravesical treatment were also comparable between groups. On multivariable logistic regression, three factors emerged as independent predictors of pathological upstaging: variant histology (primarily squamous differentiation; *p* = 0.003), urethral involvement (*p* = 0.003), and concomitant CIS (*p* = 0.042). The absence of a significant univariate difference in CIS prevalence between groups (65.6% vs. 76.0%, *p* = 0.84) does not contradict this finding; when variant histology and urethral involvement are held constant, the independent contribution of CIS to upstaging risk becomes apparent. Referral from an external institution was also identified as an independent predictor, possibly reflecting variability in TURBT quality or pathological review.

### 3.4. Upstaged NMIBC vs. Primary MIBC: Pathological and Clinical Features

Comparing the upstaged NMIBC group with those presenting with primary MIBC, both groups were well matched for age, BMI, ASA score, and surgical approach. There were no significant differences in the median number of lymph nodes retrieved (16.3 ± 8.5 vs. 16.0 ± 9.6). The rate of pathologically node-positive disease was 24.2% in the upstaged group (24/99 evaluable patients) compared with 28.7% in the primary MIBC group (*p* = 0.48). Of note, no patient in the upstaged group was classified as upstaged solely on the basis of nodal positivity; all 102 met the pT ≥ 2 criterion at final pathology. Variant histology rates and ureteric involvement were similarly distributed between groups.

The final pathological stage distribution differed significantly: upstaged patients more frequently had pT2 disease (47.1% vs. 21.9%, *p* < 0.001), while pT3 rates were comparable (42.2% vs. 46.9%). Urethral involvement was significantly more common among upstaged patients (20.0% vs. 9.6%, *p* = 0.003). CIS was more prevalent in the upstaged group than in the primary MIBC group (65.6% vs. 46.8%, *p* = 0.01), consistent with the high-risk biological profile of those NMIBC tumours that harboured occult muscle invasion (Table 4).

### 3.5. Survival Analysis

**Figure 1:**
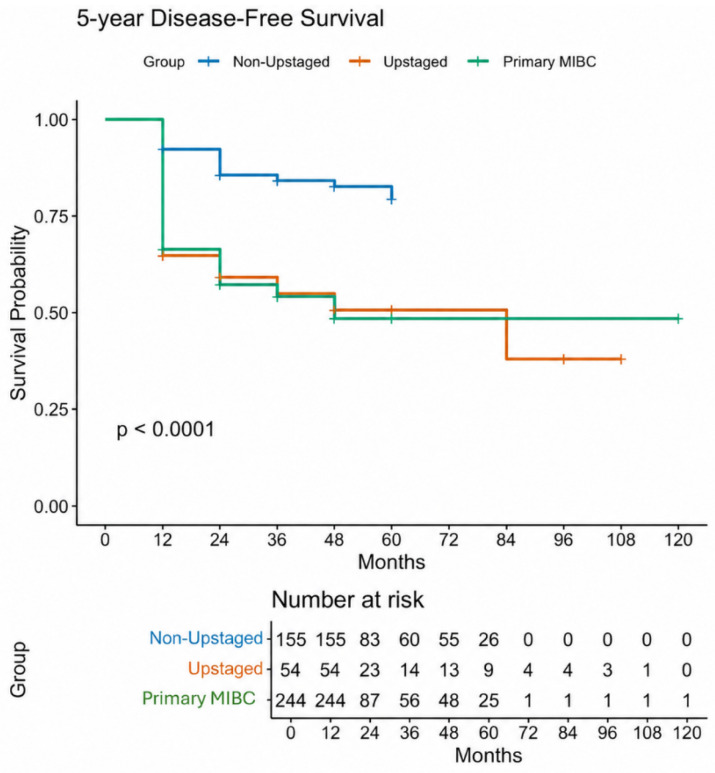
Kaplan–Meier curves for 5-year recurrence-free survival (RFS) stratified by clinical group: non-upstaged NMIBC (blue), upstaged NMIBC (red), and primary MIBC (green). Numbers at risk are shown below the x-axis at each time point. Groups were compared using the log-rank test.

**Figure 2:**
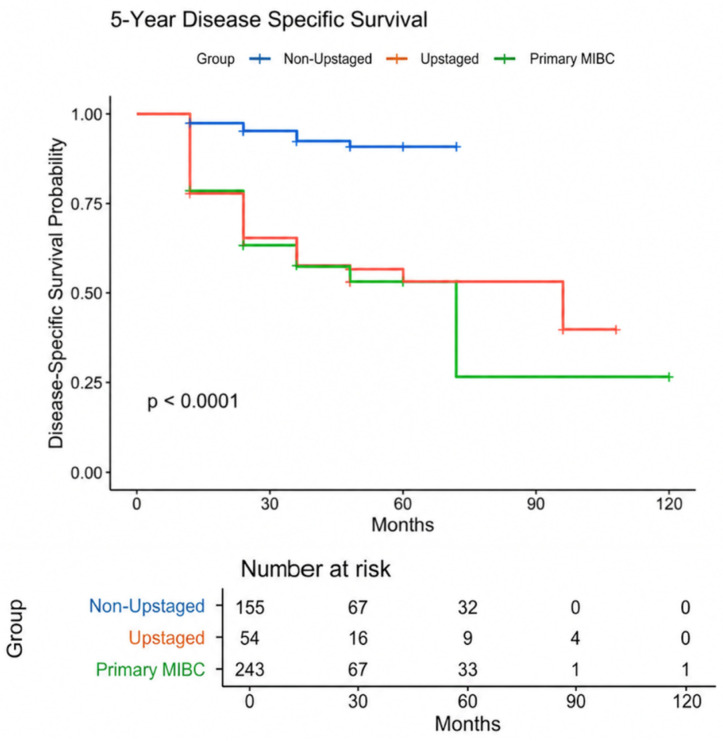
Kaplan–Meier curves for 5-year disease-specific survival (DSS) for non-upstaged NMIBC (blue), upstaged NMIBC (red), and primary MIBC (green). DSS differed significantly between non-upstaged NMIBC and both the upstaged and primary MIBC groups (log-rank *p* = 0.01 and *p* < 0.001, respectively), but not between upstaged NMIBC and primary MIBC (*p* = 0.97).

**Figure 3:**
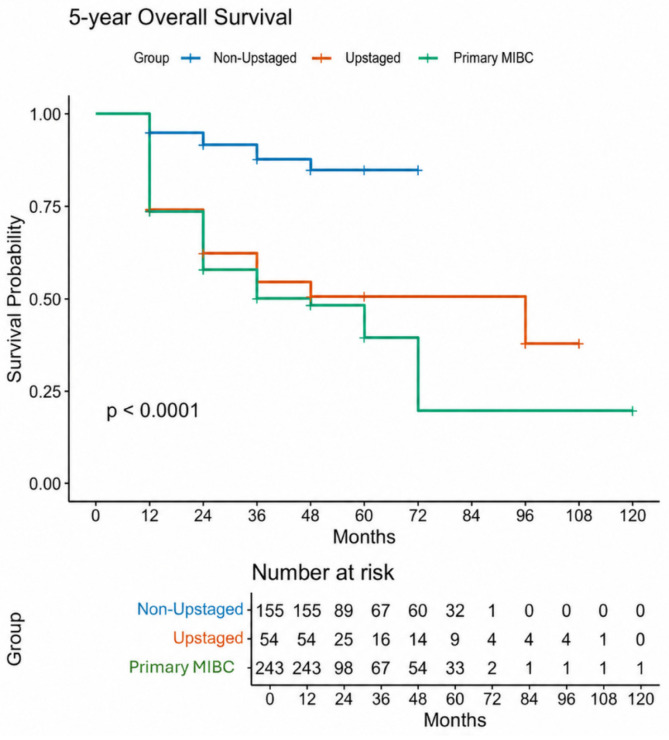
Kaplan–Meier curves for 5-year overall survival (OS) for non-upstaged NMIBC (blue), upstaged NMIBC (red), and primary MIBC (green). OS was significantly worse in upstaged NMIBC patients compared with non-upstaged patients (HR 4.64, 95% CI 2.42–8.90, *p* < 0.001) but did not differ significantly from primary MIBC patients (*p* = 0.49).

Kaplan–Meier analysis demonstrated that upstaged patients had significantly worse survival than non-upstaged NMIBC patients across all three endpoints (Figure 1, Figure 2 and Figure 3). At five years, RFS was substantially inferior in the upstaged group (log-rank *p* < 0.001), as was OS (log-rank *p* < 0.001) and DSS (log-rank *p* = 0.01). There were no statistically significant differences in 5-year RFS, DSS, or OS between upstaged NMIBC patients and those with primary MIBC, confirming that upstaging carries a survival burden equivalent to overt muscle-invasive disease. On multivariable Cox regression, upstaging was independently associated with significantly worse RFS, DSS, and OS compared with non-upstaged NMIBC patients; full hazard ratios and confidence intervals are presented in Table 5. Among additional covariates, older age and higher ASA score were independently associated with worse OS.

Multivariable Cox regression analysis confirmed these findings (Table 5). Upstaging was independently associated with worse RFS (HR 3.80, 95% CI 2.10–6.88, *p* < 0.001), DSS, and OS (HR 4.64, 95% CI 2.42–8.90, *p* < 0.001). Among other covariates, older age (HR 1.08 per year, 95% CI 1.00–1.15) and higher ASA score (HR 2.40, 95% CI 1.10–5.40) were independently associated with worse OS.

## 4. Discussion

This study analyses pathological upstaging from NMIBC to MIBC in a large, contemporary RC cohort managed over 14 years at a single tertiary centre. Upstaging occurred in more than a quarter of patients undergoing RC for NMIBC, a rate consistent with several published multicentre series [16,20]. Concomitant CIS, variant histology (particularly squamous differentiation), and urethral involvement were the strongest independent predictors of this outcome. The survival of upstaged patients was statistically indistinguishable from that of patients with primary MIBC, making clear the cost of preoperative understaging.

The 27% upstaging rate observed in our cohort aligns well with the contemporary literature. In the largest multicentre robotic series to date—the IRCC database (n = 463)—Tillu and colleagues reported an overall upstaging rate across the study period, with older age, cT1 disease, and hydronephrosis emerging as multivariate predictors [16]. Our data did not identify hydronephrosis as an independent predictor, possibly reflecting selection bias inherent to an NMIBC cohort, in which hydronephrosis is a rarer finding than in MIBC series. A separate RARC cohort (n = 277) by Jaime-Casas et al. confirmed that hydronephrosis conferred the highest odds of upstaging and that upstaged patients—irrespective of NAC receipt—had significantly worse OS [17]. The consistency of these findings across different institutional settings and surgical approaches strengthens their clinical relevance.

CIS emerged as an independent predictor of upstaging on multivariable analysis, even though its prevalence did not differ significantly between upstaged and non-upstaged NMIBC groups on univariate analysis (65.6% vs. 76.0%, *p* = 0.84). This likely reflects the interaction between CIS and other high-risk features in the multivariable model—particularly variant histology and urethral involvement—rather than a simple univariate association. When these covariates are held constant, the independent contribution of CIS to upstaging risk becomes apparent, a pattern well recognised in multivariate modelling of correlated clinicopathological variables. CIS was, however, significantly more prevalent in the upstaged group than in the primary MIBC group (65.6% vs. 46.8%, *p* = 0.01), consistent with its known field-effect nature and its role as a marker of broad urothelial genomic instability that may harbour occult invasion despite a superficial endoscopic appearance [21].

Variant histology was the strongest predictor of upstaging in our cohort (*p* = 0.003), consistent with multiple prior reports. The AUA/SUO 2024 guideline amendment specifically acknowledges the high upstaging risk associated with variant histology and recommends consideration of early RC in patients presenting with non-muscle-invasive urothelial carcinoma exhibiting variant differentiation [12]. The EAU 2025 MIBC guidelines further stipulate that micropapillary variant T1 high-grade disease should be treated with immediate RC and lymphadenectomy, regardless of BCG response [13]. In our series, squamous differentiation was the predominant variant, accounting for 7.0% of the 698 specimens with recorded histology. At cystectomy, patients with variant histology were significantly more likely to harbour pT3–T4 disease than those with pure urothelial carcinoma, consistent with findings from long-term multicentre RARC analyses in which squamous differentiation carried an OR of 6.6 for upstaging [16].

Urethral involvement was present in 20.0% of upstaged patients versus just 3.3% of non-upstaged NMIBC patients (*p* = 0.003). Prostatic urethral CIS or ductal extension indicates broad urothelial field change and has been identified as a prognostic factor for recurrence and disease-specific mortality in T1 high-grade disease treated with BCG [22]. Published data from Farré and colleagues similarly identified prostatic urethral involvement and pT1 with concomitant CIS as the most important predictors of upstaging at cystectomy in BCG-treated patients [23]. Taken together, these observations support routine mapping biopsies of the prostatic urethra in patients with high-risk NMIBC prior to definitive management decisions.

The demographic associations between female sex and Black ethnicity, with upstaging on univariate analysis, merit careful interpretation. Several studies have reported that women with bladder cancer tend to present at more advanced stages than men, possibly because haematuria in women is more likely to be attributed to benign causes, leading to diagnostic delay [24]. The association with Black ethnicity is less well characterised, and may reflect a combination of socioeconomic factors, differential access to TURBT quality, and biological differences in tumour biology—though our cohort size limits firm conclusions [25]. These associations did not remain independently significant on multivariable analysis, consistent with the hypothesis that they represent confounders of tumour biology rather than independent biological drivers of upstaging.

Nevertheless, these associations warrant attention at a systems level. Delayed diagnosis of haematuria in women and documented inequities in access to high-quality TURBT among Black patients may both contribute to a higher burden of occult muscle invasion at the time of cystectomy. Prospective studies with granular data on diagnostic delay and referral pathways are needed to disentangle biological from socioeconomic contributors.

The survival equivalence between upstaged patients and those with primary MIBC carries important practical implications. It suggests that the window of opportunity to improve outcomes through perioperative interventions—most critically, NAC—is effectively lost once upstaging has occurred. By the time the patient is found to have pT2 disease at cystectomy, the chance to administer cisplatin-based NAC (which confers a 5–8% absolute survival benefit in cT2–4a disease) has already passed [3,4]. In our cohort, 122 of 448 primary MIBC patients (27.2%) received NAC, and a subgroup analysis did not demonstrate a statistically significant survival difference between those who did and did not receive NAC. This finding should be interpreted cautiously, given the retrospective, non-randomised nature of the comparison and potential selection effects; nevertheless, it does not undermine the principal argument that upstaged patients are deprived of the opportunity for guideline-recommended NAC at the point when it would be most appropriate. This reinforces the argument for risk -tratification-guided early cystectomy in patients with high-risk features, even before BCG failure, as advocated by both EAU and AUA guidelines [11,12].

Two complementary strategies may help reduce the rate of upstaging: improving preoperative staging accuracy and identifying patients at high risk of harbouring occult muscle invasion who should proceed directly to RC. Vesical MRI using the VI-RADS scoring system has demonstrated pa pooled sensitivity of 0.92 and aspecificity of 0.88 for distinguishing NMIBC from MIBC in systematic reviews, though prospective real-world accuracy may be lower [26]. Molecular subtyping using genomic classifiers such as Decipher Bladder offers an additional avenue: non-luminal tumours exhibit significantly higher rates of upstaging to MIBC than luminal counterparts (51% vs. 32%, *p* = 0.03), and this difference is maintained on multivariable analysis [19]. Integration of these tools into clinical practice may allow a more personalised approach to the NMIBC-to-cystectomy pathway.

This study has several limitations. The retrospective single-institution design introduces inherent selection bias, and although data were prospectively maintained, some clinical variables—including the precise extent of intravesical therapy, re-TURBT indications, and full imaging details—were not uniformly captured. There was no centralised pathological re-review of all cases, which may introduce variability in the identification of histological variants or the assignment of CIS. Although NAC data were available for the primary MIBC cohort—and a subgroup analysis found no significant survival difference between NAC-treated and non-NAC-treated MIBC patients—adjuvant chemotherapy data were not systematically captured across the full cohort. This precludes an assessment of whether perioperative systemic therapy influenced survival in upstaged patients who were found to have pT3 or pN+ disease at final pathology, and is a limitation of the survival comparisons presented. The relatively short median follow-up (24 months) may limit the maturity of long-term survival estimates, and DSS comparisons between groups should be interpreted with appropriate caution. Re-TURBT was performed at the surgeon’s discretion rather than as a mandated protocol, which may have introduced variability in preoperative staging accuracy. Prospective studies incorporating standardised re-TURBT protocols, systematic MRI staging, and molecular subtyping would be needed to validate and extend these findings.

## 5. Conclusions

Pathological upstaging from NMIBC to MIBC occurs in approximately one in four patients undergoing RC for clinically non-invasive disease, and is independently predicted by concomitant CIS, variant histology (particularly squamous differentiation), and urethral involvement. Upstaged patients face markedly worse oncological outcomes than those who remain NMIBC, and their survival is statistically indistinguishable from that of patients presenting with primary MIBC. This equivalence reflects the real cost of preoperative understaging: a missed opportunity for NAC, delayed definitive surgery, and a survival disadvantage that cannot be recovered postoperatively. Clinicians should apply heightened scrutiny to patients with any of the three independent risk factors identified herein, consider early RC ahead of further conservative measures, and ensure appropriate counselling regarding the genuine risk of muscle invasion at final pathology.

## Figures and Tables

**Table 1:** Pathological staging distribution, histological subtype frequency, and associated pathological features across the entire cohort (N = 826).

Pathological Feature	N	%
***Pathological T-Stage a***		
pT0/pTx	119	15.2%
pTa	63	8.1%
pTis (CIS)	93	11.9%
pT1	100	12.8%
pT2	128	16.4%
pT3	222	28.4%
pT4	57	7.3%
***Histological Subtype b***		
Pure urothelial carcinoma	590	84.5%
Squamous differentiation	49	7.0%
Sarcomatoid	14	2.0%
Micropapillary	13	1.9%
Glandular	13	1.9%
Adenocarcinoma	4	0.6%
Small cell/neuroendocrine	10	1.4%
Plasmacytoid	5	0.7%
***Associated Pathological Features***		
Concomitant CIS	286	34.6%
Positive surgical margins	56	6.8%
Ureteric invasion	56	6.8%

a: Percentages for pathological T-stage are calculated from 782 patients with complete pathological staging data; 44 patients had incomplete T-stage assignment. b: Percentages for histological subtype are calculated from 698 patients with recorded histology data. Abbreviations: CIS, carcinoma in situ.

**Table 2:** Baseline characteristics compared between NMIBC and primary MIBC groups prior to radical cystectomy.

Variable	NMIBC (n = 378)	MIBC (n = 448)
***Demographics***		
Age at diagnosis, yrs (IQR)	68.9 (12.7)	69.6 (13.1)
Age at cystectomy, yrs (IQR)	70.0 (12.9)	70.0 (13.0)
Male, n (%)	265 (70.1%)	334 (75.1%)
Female, n (%)	113 (29.9%)	111 (24.9%)
***Ethnicity***		
White/Caucasian, n (%)	260 (81.8%)	341 (87.0%)
Asian, n (%)	10 (3.1%)	8 (2.0%)
Black, n (%)	19 (6.0%)	16 (4.1%)
Mixed, n (%)	2 (0.6%)	2 (0.5%)
Other, n (%)	27 (8.5%)	25 (6.4%)
***ASA Score***		
ASA 1, n (%)	38 (13.9%)	34 (11.6%)
ASA 2, n (%)	165 (60.2%)	161 (55.1%)
ASA 3, n (%)	69 (25.2%)	95 (32.5%)
ASA 4, n (%)	2 (0.7%)	2 (0.7%)
***Preoperative Characteristics***		
Robotic-assisted RC, n (%)	272 (72.4%)	306 (68.3%)
Preoperative MRI, n (%) *	95 (25.2%)	191 (42.7%)
Referral from external unit, n (%)	246 (65.2%)	300 (67.1%)
Concomitant CIS, n (%) **	280 (74.1%)	210 (46.9%)
Variant histology, n (%) **	34 (9.0%)	93 (20.8%)

* *p* < 0.01; ** *p* < 0.001 (two-sided chi-squared or Fisher’s exact test). Abbreviations: ASA, American Society of Anaesthesiologists; CIS, carcinoma in situ; RC, radical cystectomy; IQR, interquartile range; MRI, magnetic resonance imaging.

**Table 3:** Comparison of clinicopathological features between upstaged and non-upstaged NMIBC patients.

Variable	Upstaged (n = 102)	Non-Upstaged (n = 276)	*p*-Value
***Demographics***			
Female sex, n (%)	37 (36.3%)	69 (25.0%)	0.02
Black ethnicity, n (%)	9 (9.2%)	13 (4.7%)	<0.05
***Tumour Characteristics***			
Variant histology, n (%)	21 (20.6%)	9 (3.3%)	<0.01
Concomitant CIS, n (%)	67 (65.6%)	210 (76.0%)	0.84
Urethral involvement, n (%)	20 (20.0%)	9 (3.3%)	<0.01
cT1 disease (vs. cTa), n (%)	71 (69.6%)	152 (55.1%)	0.013
Prior intravesical therapy, n (%)	61 (59.8%)	166 (60.1%)	NS
***Final Pathological Stage (Upstaged Group Only)***			
pT2, n (%)	48 (47.1%)	-	-
pT3, n (%)	43 (42.2%)	-	-
pT4, n (%)	11 (10.8%)	-	-

Abbreviations: CIS, carcinoma in situ; NS, not significant. *p*-values derived from chi-squared or Fisher’s exact test as appropriate. Final pathological stage is reported for the upstaged group only, as non-upstaged patients by definition did not reach ≥pT2.

**Table 4:** Final pathological stage distribution in upstaged NMIBC and primary MIBC patients.

Variable	Upstaged (n = 102)	MIBC (n = 448)
pT0 (%)	-	52 (10.7%)
pT2, n (%)	48 (47.1%)	98 (21.9%)
pT3, n (%)	43 (42.2%)	210 (46.9%)
pT4, n (%)	11 (10.8%)	56 (12.5%)

Remaining patients had missing data or Tx (tumour stage not evaluable).

**Table 5:** Multivariable Cox proportional hazards regression analysis for recurrence-free survival, disease-specific survival, and overall survival.

Comparison	HR	95% CI	*p*-Value
***Recurrence-Free Survival***			
Upstaged vs. non-upstaged NMIBC	3.80	2.10–6.88	<0.001
Non-upstaged vs. primary MIBC	0.26	0.16–0.41	<0.001
Upstaged vs. primary MIBC	0.99	0.63–1.55	0.97
***Disease-Specific Survival***			
Upstaged vs. non-upstaged NMIBC	2.95	1.32–6.6	***p*** ** = 0.01**
Non-upstaged vs. primary MIBC	0.14	0.07–0.27	< 0.001
Upstaged vs. primary MIBC	0.99	0.60–1.63	0.97
***Overall Survival***			
Upstaged vs. non-upstaged NMIBC	4.64	2.42–8.90	< 0.001
Non-upstaged vs. primary MIBC	0.19	0.11–0.32	< 0.001
Upstaged vs. primary MIBC	0.85	0.53–1.36	0.49
***Additional Covariates (Overall Survival)***			
Age (per year)	1.08	1.00–1.15	0.04
High ASA score (3–4)	2.40	1.10–5.40	0.03

Abbreviations: HR, hazard ratio; CI, confidence interval; MIBC, muscle-invasive bladder cancer; NMIBC, non-muscle-invasive bladder cancer; ASA, American Society of Anaesthesiologists. Reference category for all survival comparisons: non-upstaged NMIBC.

## Data Availability

The data presented in this study are available from the corresponding author upon reasonable request.
